# What Are the Results of Limb Salvage Surgery for Primary Malignant Bone Tumor in the Forearm?

**DOI:** 10.3389/fonc.2022.822983

**Published:** 2022-04-28

**Authors:** Weifeng Liu, Yongkun Yang, Tao Jin, Yang Sun, Yuan Li, Lin Hao, Qing Zhang, Xiaohui Niu

**Affiliations:** ^1^ Department of Orthopaedic Oncology Surgery, Beijing Jishuitan Hospital, Peking University, Beijing, China; ^2^ Fourth Medical College of Peking University, Beijing, China

**Keywords:** forearm, sarcoma, limb salvage, recurrence, metastasis, prognosis

## Abstract

**Background and Objectives:**

After diagnosing a primary bone tumor involving the forearm, various excision strategies and reconstruction methods must be considered. This study explored the oncological and functional outcomes of limb salvage surgery for primary malignant bone tumors in the forearm.

**Methods:**

Patients with primary forearm bone tumors (n = 369) were retrospectively analyzed between 2000 and 2017. There were 266 patients with radial tumors, and 46 (17.3%) were malignant, whereas 103 patients had ulnar lesions and 22 (21.4%) were malignant tumors. The oncological results, prognostic factors, and functional results after limb salvage surgery of forearm malignancies were analyzed.

**Results:**

The follow-up averaged 72.1 (7–192, median 62.5) months. Fifty-six patients who received limb salvage surgery were included in the final evaluation. Radius resection was performed in 38 patients, and distal radius (25 patients) was most frequent. Ulnar resection was performed in 18 patients, and the proximal ulna (13 patients) was most frequent. The surgical margins obtained were intralesional in 3 patients, marginal in 8 patients and wide in 45 patients. Local recurrence occurred in 11 patients (19.6%), and distant metastasis occurred in 14 patients (25%). The 5-year recurrence-free survival rate was 79.8%. Unplanned excision, ulnar involvement, proximal forearm location and inadequate surgical margins were associated with recurrence. The overall 5-year and 10-year survival rates were 83.5 and 71.7%, respectively. Distant metastasis was a poor prognostic factor for the survival rate. Forty-two patients were evaluated by MSTS score with an average of 27.9 ± 1.5.

**Conclusions:**

The incidence of radial malignant tumors is higher than that of ulnar lesions. The distal radius and the proximal ulna are the most frequently involved sites. Unplanned excisions, ulnar tumors, proximal forearm tumors, and inadequate surgical margin are the risk factors for local recurrence. Distant metastasis is an independent poor prognostic factor of death. The oncology control and functional results of limb salvage surgery were satisfactory.

## Introduction

Primary bone tumors arising from the ulna and radius are rare compared with soft tissue tumors (1). Benign bone tumors accounted for most of the forearm tumors. Therefore, according to the general definition a disease is considered rare when it affects fewer than 1 in 2,000 people (2), the location of forearm accounted for 1–2% in all primary malignant bone tumors and surgical treatment is more challenging (3). Many tendons in the forearm are responsible for fine movement of the hand, and tumors often involve essential structures in this narrow space. As a result, the hand function will be significantly reduced after wide resection of the tumors.

Muramatsu (4) suggested the key for local control with forearm tumors was the safe surgical margin. A surgical margin of 5 cm in other sites is easily achieved, but it is challenging in the forearm. The reconstruction following tumor resection is also controversial, with three main problems: (1) some sarcomas are difficult to remove safely; (2) the defects and methods of reconstruction are varied, requiring individual design, and (3) the oncological evaluation and functional assessment need long-term follow up. How could we draw the appropriate surgical treatment strategies, it is urgently necessary to accumulate evidence-based evidence for these rare tumors.

This study included forearm primary malignant bone tumors to clarify (1) the epidemiological characteristics of primary malignant bone tumors in the forearm; (2) the oncological results and related risk factors; and (3) reconstruction methods and functional results after tumor resection.

## Materials and Methods

### Inclusion and Exclusion Criteria

With institutional review board (I.R.B.) approval, all patients in this study underwent limb salvage surgery for primary sarcoma of the forearm. Inclusion criteria were (1) primary malignant tumor of radius/ulna; (2) limb salvage surgery with resection of the tumor; (3) complete imaging (X-ray, CT, and MRI) and clinical data; (4) oncology results and complications can be evaluated; (5) follow-up time was more than 12 months, or oncological events (local recurrence, distant metastasis, or death) occurred within 12 months. The exclusion criteria were (1) bone defect and reconstruction were not involved; (2) amputation; (3) no surgical treatment or rejection of treatment; (4) incomplete imaging and follow-up data.

### General Characteristics

**Figure 1:**
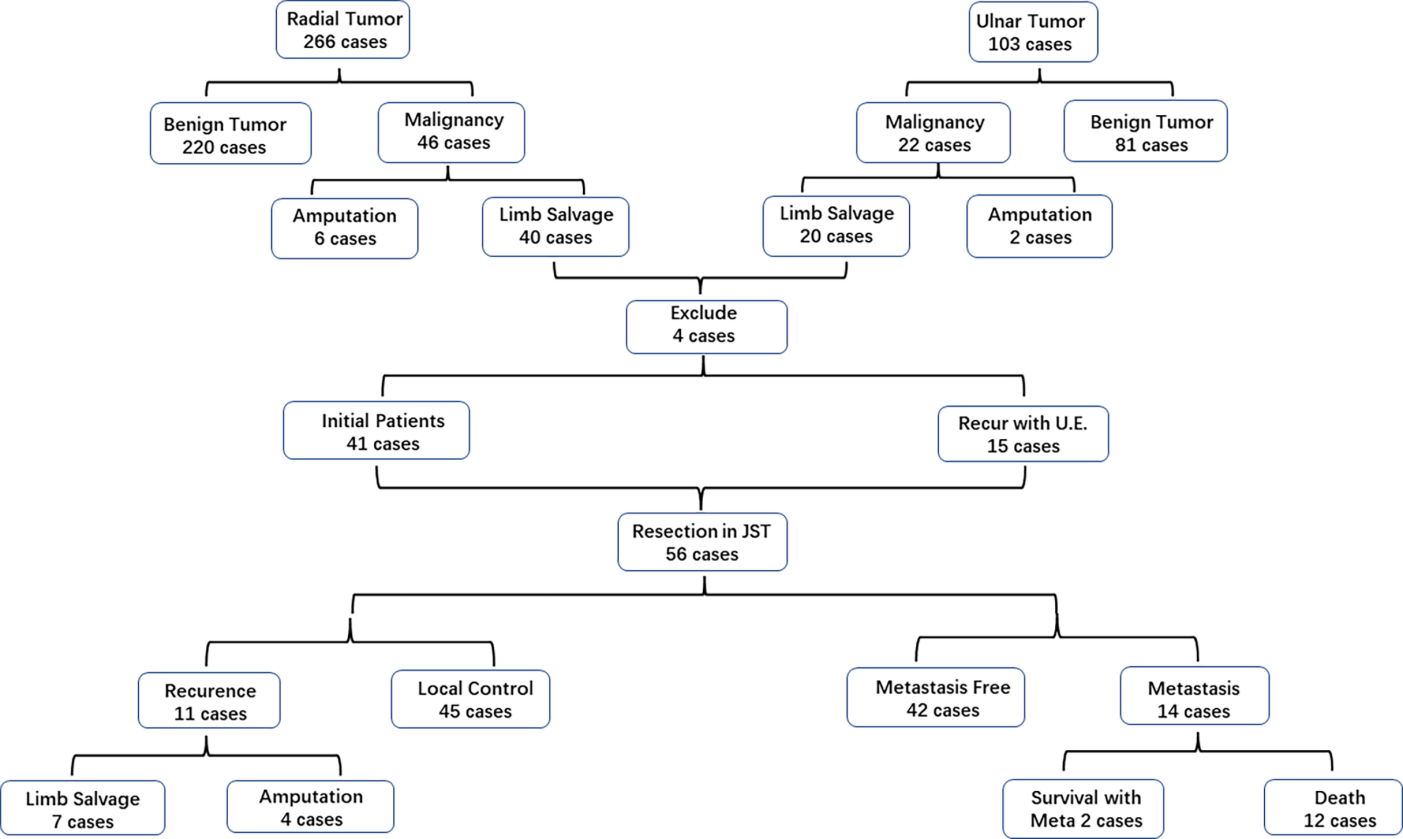
Overview of case enrollment and treatment process in this study.

Patients with primary bone tumors (n = 369) of the forearm at the Beijing Jishuitan Hospital were analyzed retrospectively. There were 266 radial tumors, and 46 patients (17.3%) had malignant lesions. Forty of these 46 patients underwent limb salvage surgery and were thus eligible for inclusion in this study. There were 103 ulnar tumors, and 22 patients (21.4%) had malignant lesions. Twenty of these 22 patients underwent limb salvage surgery and were thus eligible for inclusion. Fifty-six of these 60 eligible patients followed up for more than 12 months and enrolled in the final study (**Figure 1**).

The local evaluation included X-ray, CT, and MRI of the forearm in all patients. Staging evaluation included chest CT and bone scans. A preoperative biopsy was performed for tumors suspected of malignancy. The surgical strategy for tumor resection was based on preoperative imaging. Preoperative chemotherapy was recommended for patients younger than 55 y with high-grade sarcoma involvement.

The collected data included

1. *Surgical procedure*: All these surgical strategies were decided by the Jishuitan sarcoma multidisciplinary team with the same theory and techniques, and all the surgeons were all in our musculoskeletal tumor team. Margin was defined as follows: Intralesional: Piecemeal debulking or curettage, which may leave macroscopic disease; Marginal: Shell out en bloc through pseudocapsule or reactive zone, which may leave either “satellite” or “skip” lesions; Wide: Intracompartmental en bloc with a cuff of normal tissue, which may leave “skip” lesions, Radical: Extracompartmental en bloc entire compartment with no tumor residual (5). We elaborate on the location of the lesion in the long bone, the proportion of resection in the whole bone and the reconstructive method recorded.

High-grade malignant bone tumors contained osteosarcoma, Ewing’s sarcoma, and undifferentiated pleomorphic sarcoma received preoperative chemotherapy, which facilitates to protect the vascular nerve tract, reduced reaction zone, and is conducive to limb salvage procedure. Otherwise, limb salvage will not be performed if the response to chemotherapy is poor or if blood vessels are involved.

2. *Oncological concerns*: local relapse and recurrence-free interval, distant metastasis, and death were noted and documented in this study.
3. *Functional parameters*: the complications and MSTS (musculoskeletal tumor society) scores (6) were included in the final evaluation.

### Statistical Methods

Follow-up time was calculated from the date of operation to the last follow-up or death date. Comparison between subgroups was made using chi-square and t-tests. Wilcoxon method was used for correlation comparison of abnormal distribution grade data, with Mann–Whitney for independent samples. Local recurrence-free survival (LRFS), distant metastasis-free survival (DMFS), and overall survival (OS) were calculated using the Kaplan–Meier method. Univariate analysis for prognostic factors was performed using the log-rank test. Multivariate analyses of factors predicting outcome were performed using Cox regression. A *P*-value of 0.05 or less for two-sided comparisons was considered statistically significant. All analyses were carried out using the SPSS 21.0 software package (IBM, USA).

## Results

### Patients and Tumor Characteristics

There were forty-six patients with radial malignant tumors, accounting for 17.3% of 266 total radial tumors, and twenty-two patients with ulnar malignant tumors accounting for 21.4% of 103 total ulnar tumors.

Of the 46 patients with primary malignant bone tumor of the radius, limb salvage surgery was performed in 40 patients and amputation in 6 patients. In 22 patients with malignancy of ulna, limb salvage surgery was performed in 20 patients and amputation in 2 patients. Fifty-six patients followed up for more than 12 months, or progression within 12 months were included in the final evaluation (**Table 1**). There were 34 men (60.7%) and 22 women (39.3%) with a mean age of 27.8 (5–73, median 20.0) years. The follow-up averaged 72.1 (7–192, median 62.5) months.

**Table 1:** Patients, Tumor Characteristics and Outcomes in 56 Patients.

Characteristics	N (%)	Local recurrence	Metastasis	Death
Gender				
Male	34 (61)	8	11	9
Female	22 (39)	3	3	3
Age				
<50	48 (86)	10	12	10
≥50	8 (14)	1	2	2
Major histologic type				
Osteosarcoma	17 (30)	3	6	5
Ewing sarcoma	10 (18)	2	3	3
Pleomorphic undifferentiated sarcoma	7 (13)	1	2	2
chondrosarcoma	6 (11)	1	0	0
Other than above	16 (28)	4	3	2
Status at presentation				
Initial	41 (73)	5	9	8
Unplanned excision	15 (27)	6	5	4
Grade				
Low	17 (30)	4	2	2
High	39 (70)	7	12	10
Involved bone				
Radius	38 (68)	4	10	9
Ulna	18 (32)	7	4	3
Anatomic location				
Proximal 1/3	19 (34)	7	7	6
Middle 1/3	10 (18)	2	1	1
Distal 1/3	27 (48)	2	6	5
Bone Resection				
defect <1/3	18 (32)	3	5	4
1/3≤defect<2/3	24 (43)	6	6	5
2/3≤defect	14 (25)	2	3	3
Margin				
Intracapsular	3 (5)	2	1	1
Marginal	8 (14)	5	5	4
Wide	45 (81)	4	8	7
Chemotherapy				
Neoadjuvant	28 (50)	5	9	8
Adjuvant	33 (59)	7	10	8
No chemo	23 (41)	4	4	4

Based on the pathological diagnosis, osteosarcoma was reported in 17 patients (30.4%), Ewing’s sarcoma in 10 patients (17.9%), undifferentiated pleomorphic sarcoma in 7 patients (12.5%), low-grade central osteosarcoma in 6 patients (10.7%), chondrosarcoma in 6 patients (10.7%), bone angiosarcoma in 2 patients (3.6%), epithelioid sarcoma in 2 patients (3.6%), parosteal osteosarcoma, low-grade mixed tumor, low-grade myofibroblastic sarcoma, malignant giant cell tumor of bone, spindle cell sarcoma and clear cell sarcoma in 1 patient (1.8%), respectively. There were 17 cases (30.4%) of low-grade sarcoma and 39 cases (69.6%) of high-grade sarcoma based on histology (7, 8).

### Tumor Local Control

**Figure 2:**
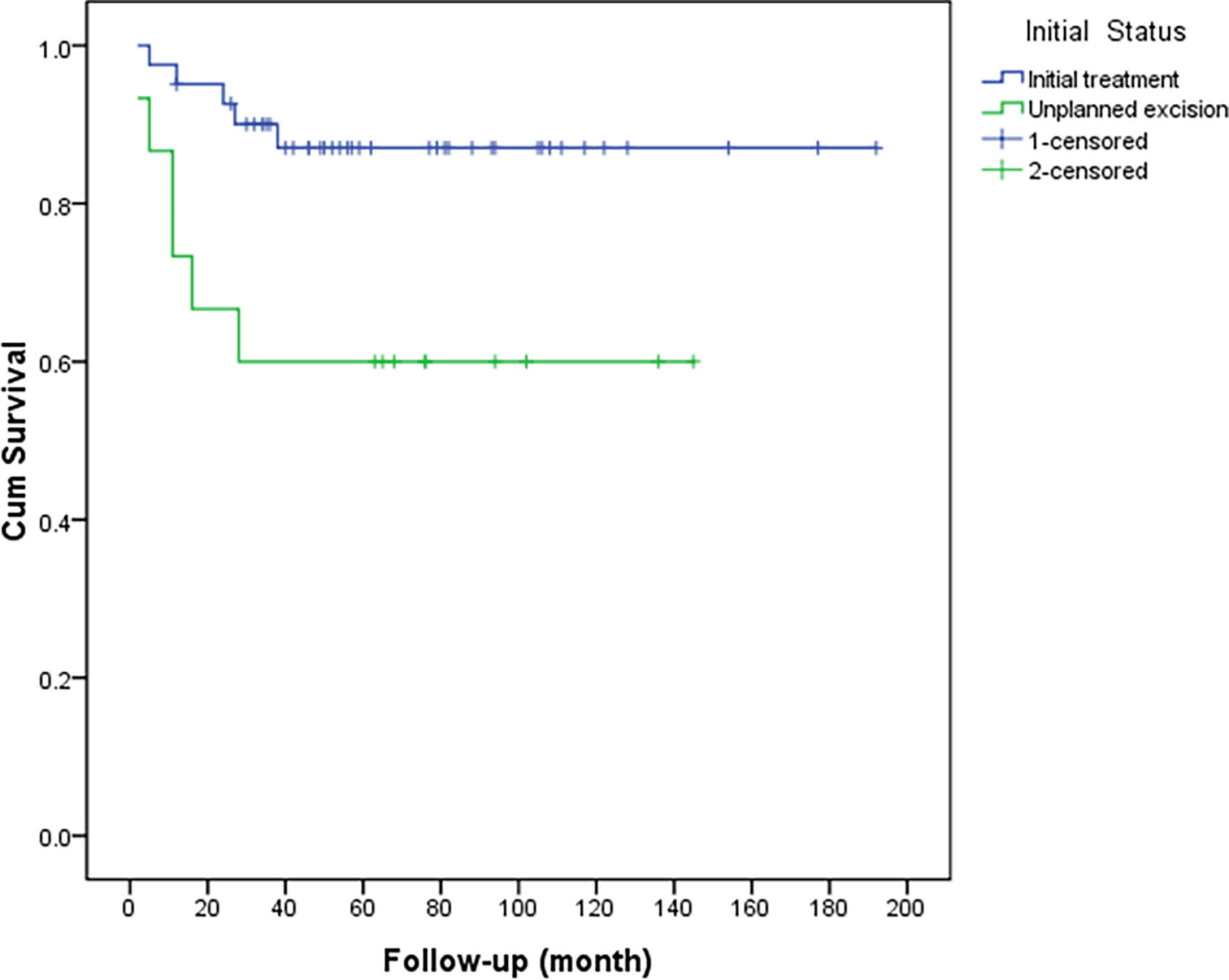
Comparison of recurrence-free survival between patients with recurrence after unplanned excision and those with initial treatment (P = 0.015).

**Figure 3:**
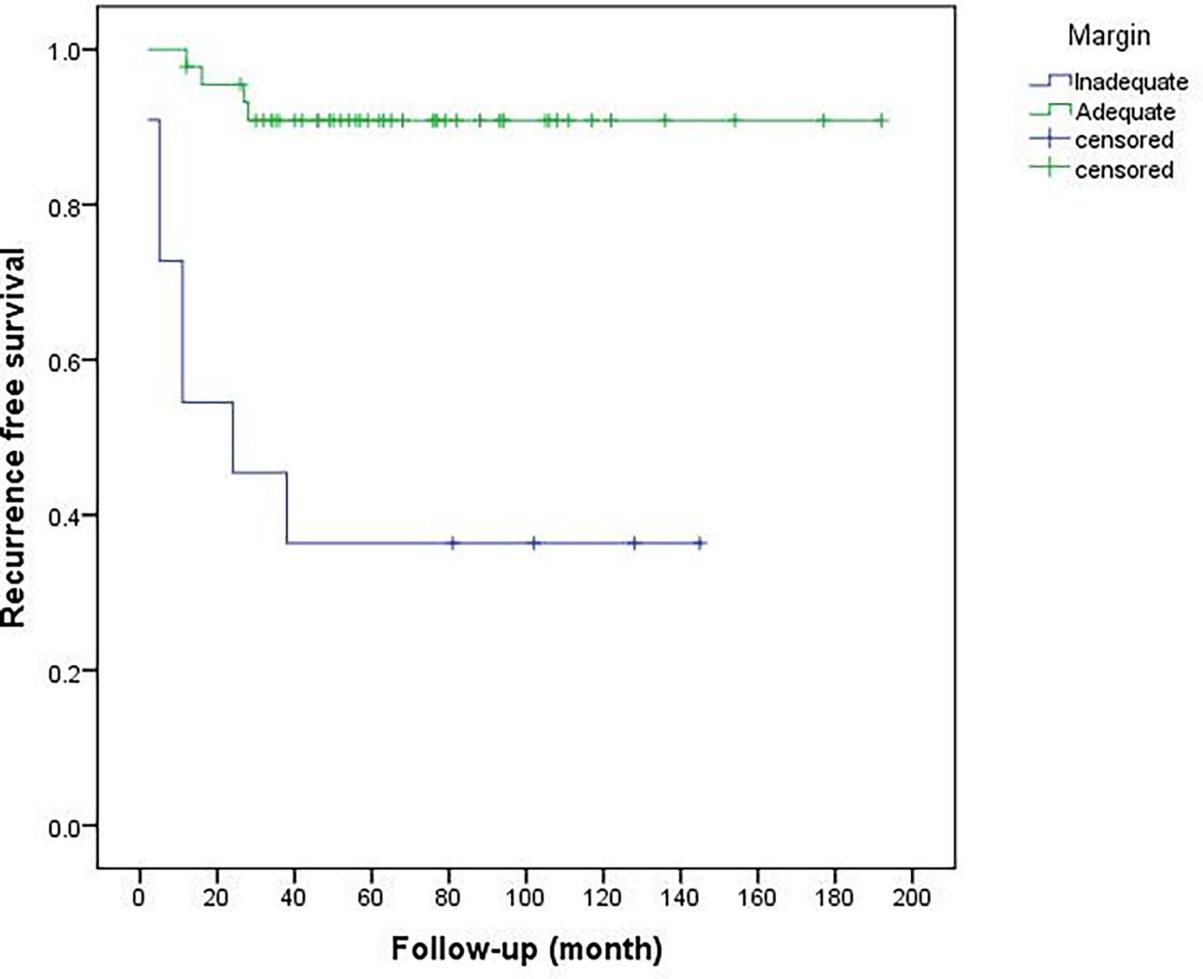
Comparison of recurrence-free survival between inadequate and adequate surgical margins (P < 0.001).

Of 56 limb salvage procedures in this study, 15 patients (15/56, 26.8%) were recurrent cases following unplanned excision (UE) in another hospital and were referred to our center with reoperation (Group 1), meanwhile, 41 patients (41/56, 73.2%) underwent initial surgery in our hospital (Group 2). In all patients of this study, local recurrence eventually occurred in 11 patients (11/56, 19.6%; see **Table 2**) at the end of follow-up after our surgery. Six patients in Group 1 (6/15, 40%) had recurrences after re-operation done at other hospitals. This is higher than the recurrence rate if the initial surgery was performed in our center (Group 2) (5/41, 12.2%) (P = 0.02) (**Figure 2**). The median recurrence-free time for these 11 recurrent cases was 12 (2–38) months, and 90% of the recurrences occurred within three years (10/11). There were 4 cases who eventually had to undergo amputations in these 11 recurrent cases (4/11, 36.4%). The local resection was performed in 7 cases (63.6%), and one case had a second recurrence. The 3-year and 5-year recurrence-free survival rates were 81.9 and 79.8%, respectively. The recurrence rate with inadequate (marginal or intralesional) margins was significantly higher than adequate (wide) resections. Univariate analysis (**Table 3**) shows the history of UE (P = 0.015), ulnar tumor (P = 0.016), tumor located in the proximal forearm (P = 0.021), and inadequate surgical margin (P <0.001) were associated with recurrence (**Figure 3**).

**Table 2:** Local Recurrences by Tumor Type, Grade, Location, Margins.

No.	Histology	Post-op interval	Grade	Bone	Location	Status	Margin	Outcome	Follow-up months
1	Osteosarcoma	2	High	Radius	Distal 1/3	Unplanned excision	Inadequate	Death	7
2	Osteosarcoma	38	High	Radius	Distal 1/3	Initial	Inadequate	NED	123
3	Spindle cell sarcoma	11	Low	Radius	Distal 1/3	Unplanned excision	Inadequate	Death	19
4	Ewing sarcoma	12	High	Ulna	Proximal 1/3	Initial	Adequate	Death	24
5	Ewing sarcoma	28	High	Ulna	Proximal 1/3	Unplanned excision	Adequate	Death	40
6	Osteosarcoma	5	High	Ulna	Middle 1/3	Initial	Inadequate	Death	92
7	Chondrosarcoma	24	Low	Ulna	Proximal 1/3	Initial	Inadequate	NED	48
8	Low grade central osteosarcoma	27	Low	Ulna	Proximal 1/3	Initial	Adequate	NED	42
9	Clear cell sarcoma	5	Low	Radius	Proximal 1/3	Unplanned excision	Inadequate	Death	11
10	Pleomorphic undifferentiated sarcoma	16	High	Ulna	Proximal 1/3	Unplanned excision	Adequate	NED	43
11	Epithelioid sarcoma	11	High	Ulna	Proximal 1/3	Unplanned excision	Inadequate	SWT	30

Inadequate, Intracapsular and Marginal; Adequate, Wide; NED, No evidence of disease; SWT, Survival with tumor.

**Table 3:** Outcomes in Univariate Analysis of Prognostic Factors (n = 56).

Variable		Local recurrence-free survival (%)	Distant metastasis‐free survival (%)	Disease specific overall survival (%)
Gender				
	Male	76.1	64.6	65.6
	Female	85.6	86.4	86.4
	P-value	0.377	0.152	0.392
Age				
	<50	78.8	74.1	74.2
	≥50	85.7	65.6	43.8
	P-value	0.620	0.986	0.609
Grade				
	Low	76.5	88.2	88.2
	High	81.4	67.0	67.7
	P-value	0.651	0.178	0.427
Bone Site				
	Radius	88.9	71.0	71.8
	Ulna	66.1	77.8	66.1
	P-value	0.016	0.762	0.662
Anatomic location				
	Proximal 1/3	62.3	51.3	48.1
	Middle & Distal 2/3	88.3	80.1	79.5
	P-value	0.021	0.119	0.065
Status				
	Initial	87.0	74.3	72.2
	Unplanned excision	60.0	66.7	72.7
	P-value	0.015	0.419	0.409
Margin				
	Adequate	90.8	80.4	80.9
	Inadequate	36.4	43.6	48.5
	P-value	0.000	0.008	0.048
Chemotherapy				
	Neoadjuvant & Adjuvant	78.2	79.1	70.8
	No chemo	82.2	68.7	72.5
	P-value	0.741	0.321	0.833
Local recurrence				
	Yes	NA	36.4	26.5
	No	NA	82.2	81.8
	P-value	NA	0.000	0.000
Metastasis				
	Yes	NA	NA	0
	No	NA	NA	100
	P-value	NA	NA	0.000

### Postoperative Complications and Functional Evaluation

**Figure 4:**
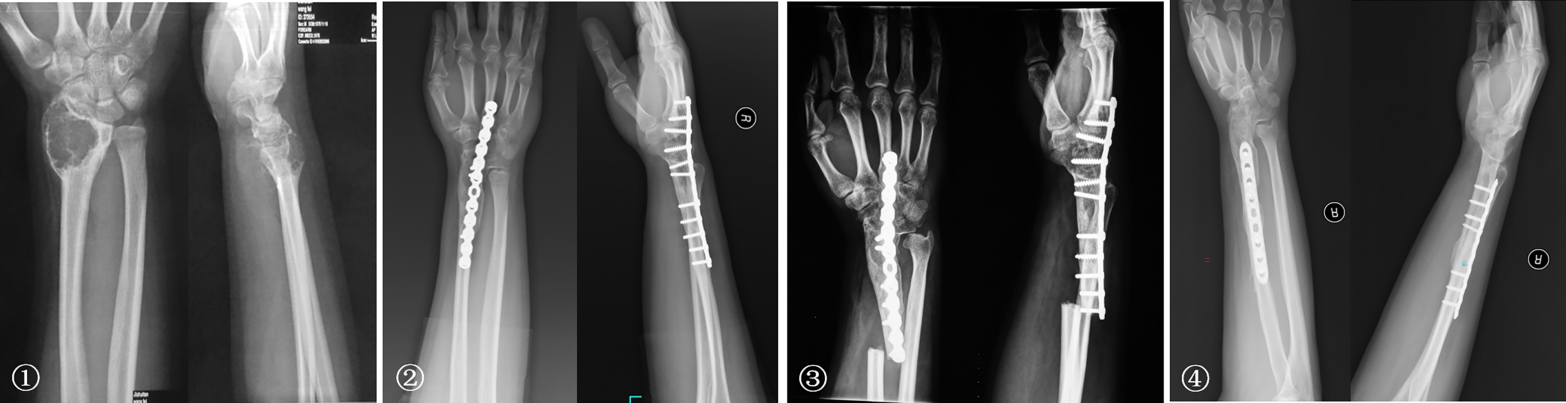
① The preoperative radiographs of a 29-year-old man with chondrosarcoma. ② Treatment included a distal radius resection and autogenous iliac bone graft with wrist joint fusion. ③ Fracture was caused by trauma eight years after surgery, and internal fixation was performed again. ④ Rotation function of the forearm is shown 192 months postoperatively.

**Figure 5:**
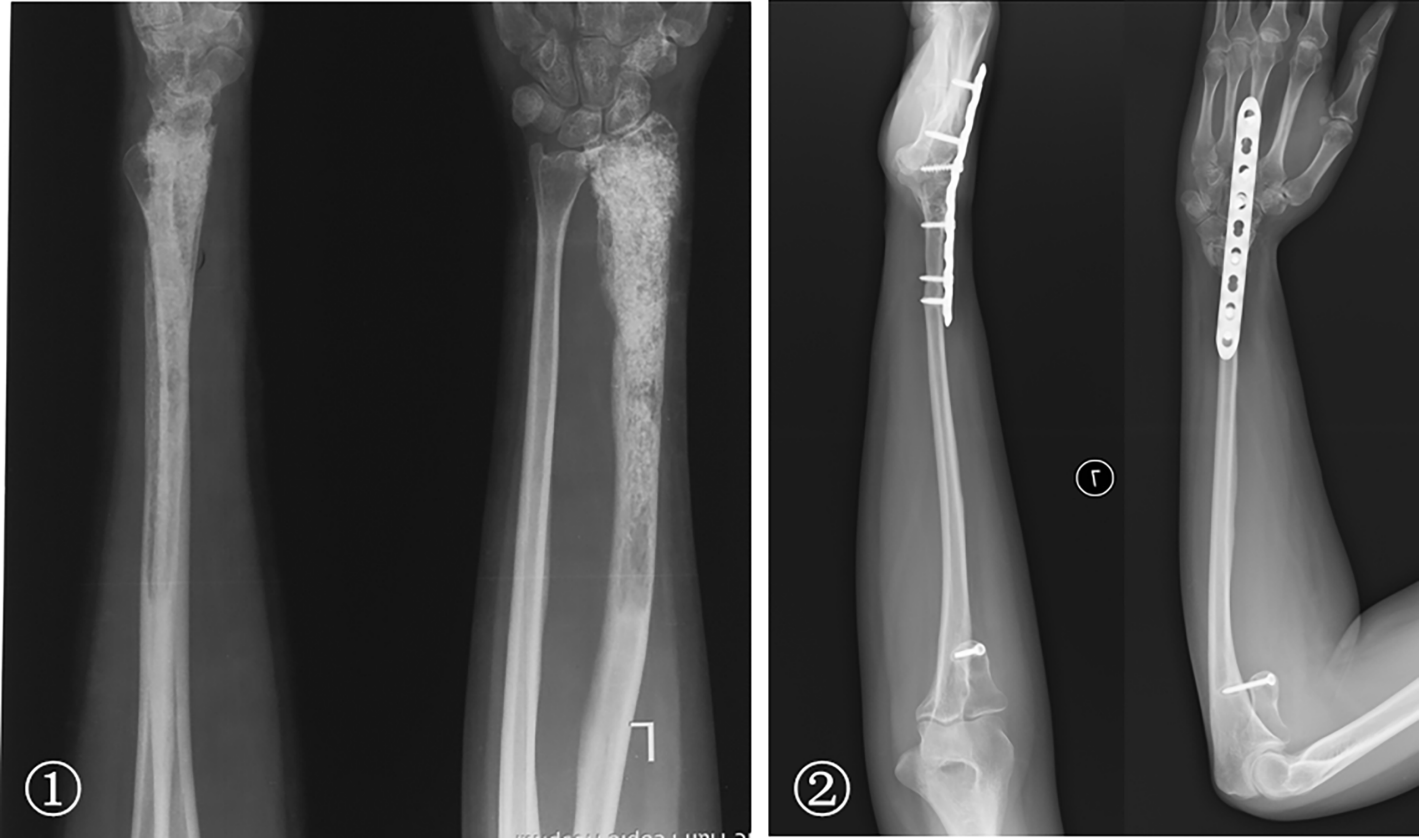
① A 29-year-old woman with low-grade central osteosarcoma of the middle and distal radius underwent unplanned excision and tumor recurrence. ② Radius resection and ulna centralization with wrist joint fusion was performed; satisfactory bone healing but a loss of forearm rotation is shown 65 months postoperatively.

**Figure 6:**
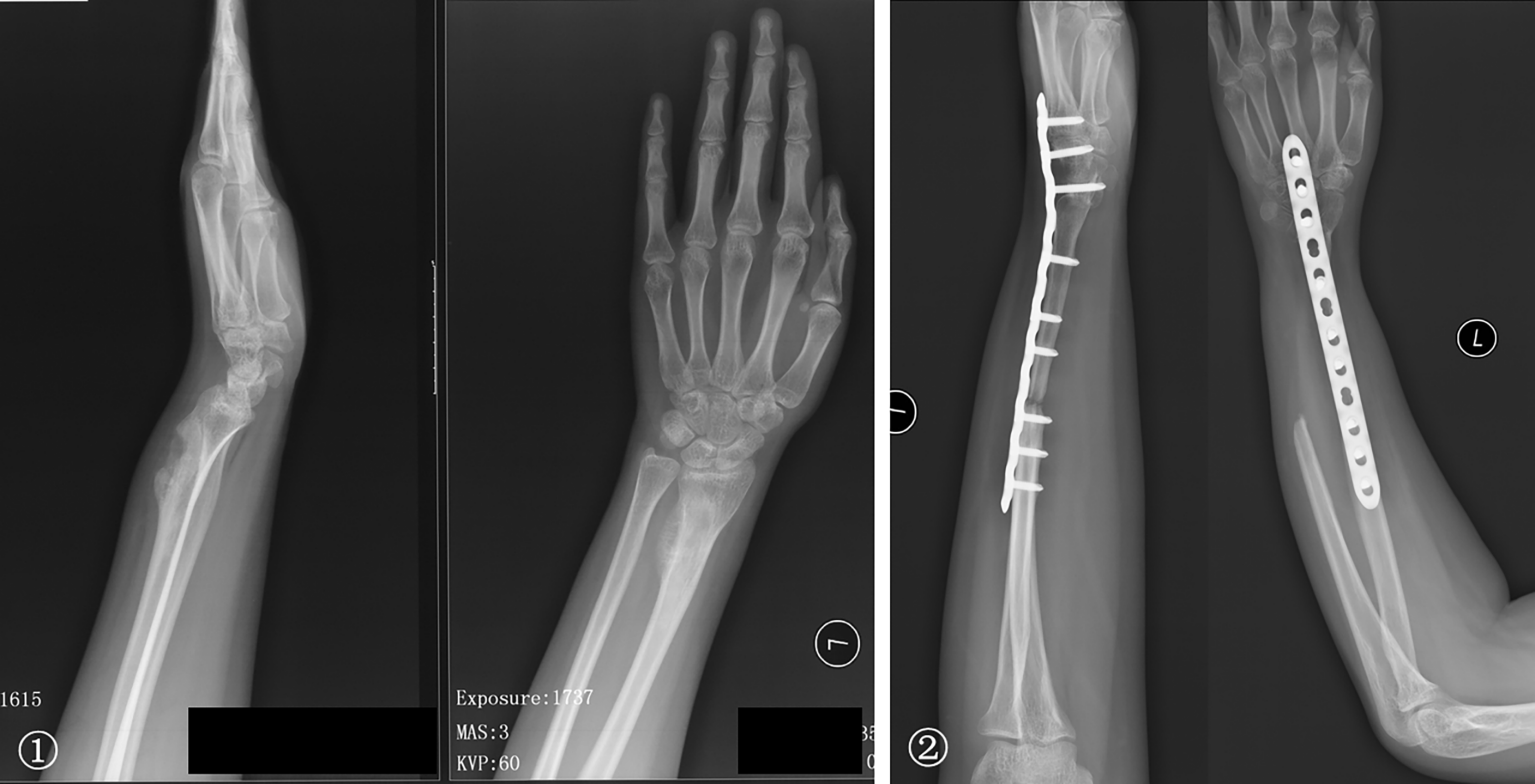
① The preoperative radiographs of a 19-year-old woman with low-grade central osteosarcoma of the distal radius. ② Treatments included a distal radius resection; ipsilateral ulnar osteotomy to replace the radial defect. Wrist joint fusion was performed; the satisfactory bone healing of the forearm and rotation function is shown 50 months postoperatively.

The bone defects after radial tumor resection were divided into proximal 1/3, distal 1/3, and more than 1/3 defect. The proximal 1/3 defect did not receive reconstruction. The distal 1/3 defect received an autogenous iliac bone graft and wrist joint fusion with internal fixation (**Figure 4**). The more than 1/3 defect from distal to proximal radius received the following procedures: (1) ulna osteotomy and fixation with the end of radius, (2) ulna centralization and wrist arthrodesis with internal fixation (**Figure 5**), (3) long segment fibula autograft and fixation (less than 1/2 defect), and (4) ipsilateral ulnar osteotomy to replace the radial defect (**Figure 6**)

**Figure 7:**
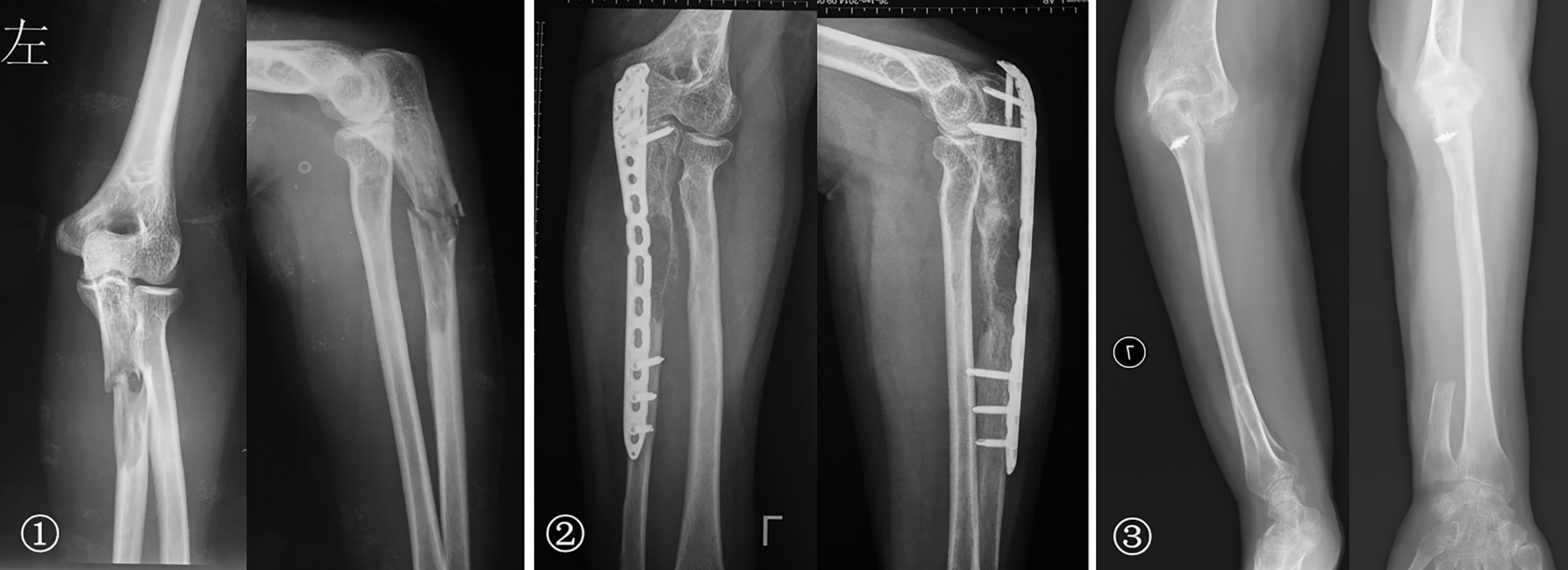
① The preoperative radiographs of a 51-year-old woman with osteosarcoma of the proximal ulna. ② An unplanned excision and tumor recurrence occurred. ③ The radial head was displaced and inserted into the intercondylar of the humerus after proximal ulna resection; the rotation function of the forearm is shown 76 months postoperatively.

After resecting the ulnar tumor, the proximal 1/3 defect was treated with (1) elbow prosthesis replacement and (2) inactivated replantation. More than 2/3 defect of the middle segment was treated with (1) elbow prosthesis combined with free vascularized fibula grafting and (2) brachioradialis elbow arthroplasty (**Figure 7**). The distal 1/3 defect did not receive reconstruction. Although we have performed different methods in reconstruction, the majority is biological reconstruction, which determined relatively few subsequent complications.

Ten patients (10/56, 17.8%) developed postoperative complications: internal fixation failure in 5 patients, limb shortening deformity, wrist silver fork deformity, prosthetic aseptic loosening, inactivated bone graft joint subluxation, and bone graft nonunion in 1 patient, respectively. Seven patients (7/10, 70%) underwent revision: 5 patients with fixation failure received re-fixation, one patient with nonunion received iliac graft again, and one patient with limb shortening deformity received limb extension by external fixator. The other three patients underwent routine observations without revision.

Twenty-two patients with ulna centralization lost rotational function, but flexion/extension and other fine movements were not significantly limited. At the final follow-up, functional scores were analyzed for both survivor and final limb salvage patients, because 12 patients died and 4 patients underwent amputation due to recurrence (2 patients were repeated), so 42 patients were included in the final functional evaluation. The MSTS score with an average of 27.9 ± 1.5. The function of patients with limb salvage was satisfactory, and the final limb salvage rate was 92.9% (52/56).

### Distant Metastasis and Overall Survival

The follow-up averaged 72.1 (7–192, median 62.5) months. None of the patients had metastatic disease at presentation and distant metastasis was observed in 14 patients (14/56, 25%) during the follow-up, there were seven osteosarcomas, three Ewing sarcomas, two undifferentiated pleomorphic sarcomas, and two low-grade central osteosarcomas developed metastatic disease, 12 (12/14, 85.7%) of them had high-grade sarcomas. The median time from surgery to the development of distant metastasis was 15 (2–64) months, with 6 (42.9%) metastases occurring within 1 year and 12 cases (85.7%) within two years. The median time from the development of distant metastases to death was 11 (1–84) months. Eleven cases (78.6%) involved only lung metastases, 3 cases (21.4%) involved multiple sites of lung and bone metastases (one scapula, one thoracic vertebra, and one femoral shaft).

The 2-year and 5-year metastasis-free survival rates were 78.6 and 76.0%, respectively. The metastasis-free survival rates with adequate (wide) margins and inadequate (marginal or intralesional) margins were 80.4 and 43.6%, respectively (P = 0.008). The 5-year survival rates of high-grade and low-grade tumors were 81.7 and 88.2%, respectively (P = 0.427).

**Figure 8:**
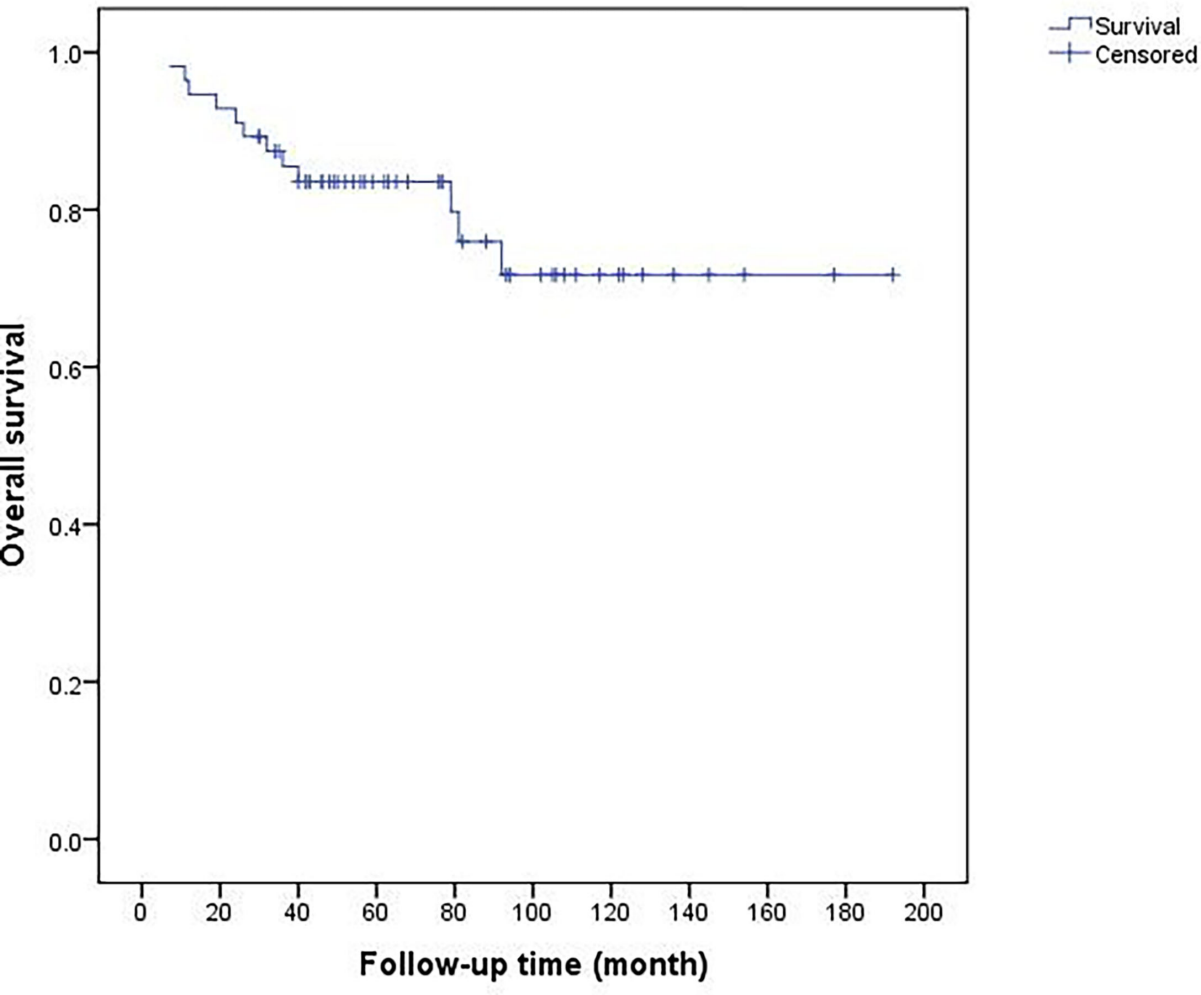
Overall 5-year and 10-year survival rates of 56 patients were 83.5 and 71.7%, respectively.

At the end of follow-up in Oct 2021, forty-two patients survived without tumor, two patients survived with metastatic disease, and twelve cases died of metastasis. The median survival time of dead patients was 29 (7–92) months. The overall 5-year and 10-year survival rates were 83.5 and 71.7%, respectively (**Figure 8**). Univariate analysis showed inadequate surgical margins (P = 0.048), local recurrences (P <0.001) and distant metastases (P <0.001) were associated with death. Multivariate analysis of the risk ratio model showed only distant metastases were significant independent poor prognostic factors of overall survival (P <0.001) (**Table 4**).

**Table 4:** Outcomes in Multivariate Analysis of Prognostic Factors.

Variable		Wald	Odds Ratio	*P*-Value	95% CI	
					Lower	Upper
Margin	Adequate	0.202	1	0.653	0.574	2.424
	Inadequate		1.180			
LR	No	0.752	1	0.386	0.484	6.529
	Yes		1.778			
Metastasis	No	13.864	1	0.000	7.006	530.539
	Yes		60.966			

LR, Local recurrence.

## Discussion

The incidence of primary malignant bone tumors of the forearm is low. Limited previous studies describe a large case series of bone tumors in the forearm, most of which are soft tissue tumors (9–11). The complex anatomy in the narrow forearm space leads to difficulties of limb salvage surgery and poor function after limb salvage surgery for treating bone sarcoma. In the forearm tumors treated in our center at the past 18 years, more benign tumors were found than malignant tumors, and more soft tissue sarcoma was found than primary bone malignant tumors (1). Many reports on soft tissue sarcoma in the forearm have been published (12), while only some case reports on bone sarcoma have been found (13, 14). The primary malignant tumors in the forearm only occupied 18.4% (68/369) of all primary bone tumors in this study. Although the number of malignant cases in the radius is much greater than that in the ulna, the proportion of malignant ulnar tumors is higher. Therefore, tumors in the ulna are much more likely to be malignant, although the number of malignant tumors in the radius is dominant. This distribution characteristic has not been described previously (15, 16).

In this study, eleven cases (11/56, 19.6%) had local recurrence in the final follow-up. Six of the 15 patients (6/15, 40%) underwent UE before recurrence developed. These factors may be relevant with the high recurrence rate: improper surgical approach, surgical field contamination, and compartment barrier destruction resulted in the spread of the tumors; the biological behavior of recurrent tumors was more aggressive (17, 18). A significant advantage in recurrence-free survival for primary tumors was observed, and their imaging findings were “milder” than those of UE tumors. Because of the high risk of recurrence, radical resection and even amputation should be considered.

Following univariate analysis, tumors located in the ulna and proximal forearm showed a significantly higher risk of local recurrence, the above characteristics were not found in previous studies (12, 19, 20). Compared with bone sarcomas, soft tissue sarcomas of the forearm have predominantly been previously reported, and which was focused on tumor size associated with recurrence (21). Bosma et al. (22) analyzed the different recurrence risks of sarcoma at different sites, and Pradhan et al. (23) compared forearm sarcoma with other sites. However, the different recurrence rates between different sites in the forearm had not previously been analyzed due to the small sample sizes of the studies.

With less soft tissue attached, the coverage is more difficult for limb salvage in the ulna. The proximal anatomical structure is more complex than the distal forearm. The radial nerve, brachial artery, attachment of muscles at the proximal forearm, and juxtaposition of the elbow joint may lead to inadequate resection margins due to the necessary preservation of essential structures. All these factors may contribute to the increase in the ulnar recurrence rate. For ulna malignancies, especially proximal involvement, the implementation of limb salvage needs to be repeatedly evaluated.

The influence of surgical margins on local recurrence has been investigated in many studies (12). Most researchers define adequate margins as wide or extra-compartmental resections. Muramatsu et al. (4) used a 2-cm margin for high-grade sarcomas and a 1-cm margin for low-grade sarcomas, achieving a satisfactory local recurrence rate of 11%. In this study, the inadequate surgical margin increased the recurrence rate significantly. Intralesional and marginal resection was 63% (7/11), while the recurrence rate of adequate margins was 8.9% (4/45). We planned the surgical strategy according to preoperative imaging, and we used the postoperative specimen and pathological slides to evaluate the surgical margin. This was consistent in most cases. Sometimes the postoperative evaluation does not reach the ideal-planned margin. Such outcomes suggest that limb salvage surgery needs to be re-evaluated if it is difficult to achieve a safe margin.

Daecke et al. (19) reported that the metastasis rate of high-grade bone sarcoma in the forearm was 24%, and the 5-year survival rate was 86.2%, which was better than that in other sites. The current study showed that 14 patients (14/56, 25%) had metastases, and the 5-year survival rate of high-grade sarcoma was 81.7%, slightly lower than that of low-grade sarcoma but without statistical significance. The data suggest that (1) lower tumor load in the forearm leads to a lower risk of metastasis than other anatomical locations are unknown, many other variables that contribute to the risk of metastasis (2), perioperative chemotherapy was performed in most high-grade sarcomas, which reduced metastatic risk.

Whether recurrence affects metastases and survival is controversial (24, 25), some studies suggested that safe margins only affect recurrence, which does not increase metastases and reduce survival (26–28). However, more studies have demonstrated the contrary result (9, 29, 30). The current study showed that margins and recurrence were significantly associated with metastasis and survival following univariate analysis. But only metastasis was an independent risk factor for death from the multivariate analysis. Perhaps recurrence causes repeated operations and prolongs tumor-bearing time, which potentially changes of tumor biological behavior and increases the risk of metastases. The overall 5-year survival rates were 83.5%, the 5-year survival rates of high-grade and low-grade tumors were 81.7 and 88.2% respectively in our study. It was better than the 5-year survival rate of 67% and similar to survival at 5-year following limb salvage surgery of 86% in other reports (19, 23). The results validated the concept of safe margins—local control—reduction of metastases—improvement of survival need more evidence to back up.

The premise of function is oncological safety. The anatomical features of two bones in the forearm have extensive influence on rotation and hand function. Since there is no weight-bearing, it is important to ensure flexibility for the forearm and hand. Defects of the distal ulna and proximal radius have little effect on function, and reconstruction is unnecessary. More challenges result from 2/3 or more defects in the middle and distal radius and 2/3 or more defects in the middle and proximal ulna. For the treatment strategy of the distal radius, wrist arthrodesis with structural iliac crest bone graft was chosen (ICBG) for the defect within 7 cm, and good results were achieved (31). For defects over 7 cm, the ulna is directly displaced to centralization. This method is practical and straightforward, but the loss of rotation is not negligible. A segment autogenous fibula transplantation or translocating the ipsilateral ulna as a vascularized autograft to reconstruct the distal radius defects were adopted (32, 33) to maintain rotation. This relatively complex method showed better function and preferred wrist arthrodesis to obtain a stable joint (31). Compared to a few joint replacement options for the distal radius (34), prosthesis replacement is a routine and applicable method for proximal ulna defects. Brachioradialis elbow arthroplasty between the proximal radius and humeral condyle was designed, yielding satisfactory function. It is challenging to cover skin defects due to extensive resections in recurrent cases. Instead, it is preferred to execute microsurgery and flap technology (16, 35). In this study, three patients received flap coverage, and two patients underwent free vascularized fibula grafting with satisfactory postoperative results.

This study has some limitations. Firstly, this is a retrospective analysis spanning 18 years, and there were homogeneity differences in the choice of chemotherapy and surgical techniques. Secondly, this is a single institution report, which lacks multiple center coordination to correct the bias in the enrollment of patients and treatment methods. Finally, this study only included limb salvage cases, which did not compare with the outcomes of amputation. Thus, the selection bias of tumor load and site led to overestimating the survival rate of patients with forearm malignancy.

## Conclusions

This study is the most extensive, single-institution case analysis of limb salvage treatment for primary malignant bone tumors in the forearm. A history of unplanned surgery, tumors located in the ulna, proximal forearm, and inadequate surgical margin are important factors leading to local recurrence. To improve local control, limb salvage should be used with caution in patients who underwent unplanned excision. Amputation may be a better choice for high-risk patients with proximally located soft tissue masses adjacent to vascular and nerve tracts. Metastasis is an independent poor prognostic factor of survival. Multidisciplinary collaboration for the systematic treatment of metastatic patients is a potentially effective way to reduce the mortality of these malignant tumors. Limb salvage surgery for malignant bone tumors of the forearm showed a high overall survival rate and relatively satisfactory functional recovery.

## Data Availability Statement

The original contributions presented in the study are included in the article/supplementary material. Further inquiries can be directed to the corresponding authors.

## Ethics Statement

The studies involving human participants were reviewed and approved by the Beijing Jishuitan Hospital. Written informed consent to participate in this study was provided by the participants’ legal guardian/next of kin.

## Author Contributions

WL and XN conceived and designed the study. WL,YY, TJ, YS, YL, LH and QZ undertook the study. WL and YY analyzed and interpreted the clinical data. WL drafted the manuscript. WL, YY and XN reviewed and edited the manuscript. All authors listed have made a substantial,direct, and intellectual contribution to the work and approved it for publication.

## Funding

This research was supported by the National Key R&D Program of China (Grant No. 2021YFC2400500), Beijing Municipal Administration of Hospitals Incubating Program (PX2021015), Beijing Natural Science Foundation (L212042), Beijing Jishuitan Hospital Elite Young Scholar Programme (XKGG202105), Chinese Society of Clinical Oncology (CSCO) Research Foundation (Y-2019GCTB-002, Y-young2019-070), National Natural Science Foundation of China (51973021).

## Conflict of Interest

The authors declare that the research was conducted in the absence of any commercial or financial relationships that could be construed as a potential conflict of interest.

## Publisher’s Note

All claims expressed in this article are solely those of the authors and do not necessarily represent those of their affiliated organizations, or those of the publisher, the editors and the reviewers. Any product that may be evaluated in this article, or claim that may be made by its manufacturer, is not guaranteed or endorsed by the publisher.

## Abbreviations

CT, Computed Tomography; MRI, Magnetic Resonance Imaging; MSTS, Musculoskeletal Tumor Society; UE, Unplanned Excision; LRFS, Local Recurrence Free Survival; DMFS, Distant Metastasis Free Survival; OS, overall survival; ICBG, Iliac Crest Bone Graft.
